# The Paediatric Ambulatory Consulting Service (PACS) program: a role for family pediatricians in the hospital emergency rooms 

**DOI:** 10.1186/s13052-016-0230-2

**Published:** 2016-02-25

**Authors:** Luigi Nigri, Ruggiero Piazzolla, Massimo Pettoello-Mantovani, Ida Giardino, Micaela Abbinante, Giovanni Gorgoni

**Affiliations:** Federazione Italiana Medici Pediatri (FIMP), Via Parigi, 11 (00185) Rome, Italy; European Paediatric Association, Union of National European Paediatric Societies and Associations (EPA-UNEPSA), Allt. center, Zimmerstraße 69, D-10117 Berlin, Germany; Residency Program in Pediatrics and Pediatric Research Center, University of Foggia, Via Pinto, 1 (71100) Foggia, Italy; Azienda Sanitaria Locale, Barletta-Andria-Trani (BT), Via Fornaci, 201 (70031) Andria, Italy; Puglia Region. Department of Health, Social Welfare and Sport, Via Gentile, 52 (70126) Bari, Italy

**Keywords:** Hospitalization, Economics

## Abstract

This paper describes the Paediatric Ambulatory Consulting Service (PACS) project, developed by ASL-BT (Azienda Sanitaria Locale, Barletta-Andria-Trani), an Italian regional Public Health Centers network, in response to the current global situation of economic distress.

PACS consist in integrating existing public health care services that are independently provided by hospitals and the Primary Care Paediatrics network. It has been developed with the aim to establish innovative yet efficient managerial solutions able to rationalize the resources not weakening the quality of services provided to the population.

## Correspondence/Findings

This paper describes the Paediatric Ambulatory Consulting Service (PACS) project. PACS was developed in 2014 in the Italian Region of Puglia, as a result of a close co-operation between the ASL-BT, a *State Local Health Centers* network (LHC) [1], and the Italian Federation of Paediatricians (Federazione Italiana Medici Pediatri, FIMP) in response to the current general situation of economic distress. Both ASL-BT and FIMP are part of the Regional post-graduate medical education network, and collaborate with the Residency Program in Paediatrics (RPP) of the University of Foggia.

PACS integrates existing public health care services, otherwise independently provided by public hospitals and Primary Care Paediatric networks. The PACS program, operated by family pediatricians working for the State network of Family Paediatrics, consists in a pediatric ambulatory consulting service, active in hospitals and providing a screening of the clinical conditions of outpatients <18 years old, before they access the Emergency Room (ER) Departments. PACS is active during the week-ends (Saturday-Sunday) and festivities, which in the Italian healthcare system are not covered by the public health services, that are usually provided by the Family Paediatrics only during the weekdays (Monday-Friday).

In Italy, like in other nations with similar public health systems, a massive turnout of patients is faced by hospital’s ERs, when the family doctors rest in accordance to their standard work contract agreements. Paediatric ER departments are usually active in Italy only in few large hospitals throughout the Nation, and in the remaining hospitals, pediatricians are generally not included in the ERs teams. Such situation depending mostly upon the need of containing the health care costs.

Typically, children are referred by the ER directly to the correspondent hospital Paediatric Units, even for cases showing not serious medical conditions, usually classified as “white” codes [2], and it often generates an incorrect hospitalization of children. This common situation represents a heavy burden for the hospital administration, due to the high mean daily unit cost of the pediatric hospitalization, usually related to the regional Diagnosis Related Groups (DRG) price list [3].

Therefore, PACS program provides a first clinical assessment and care for subjects <18 years old whose tutors claim the existence of a pathologic condition when turning out to the hospital ER Departments. The patients classified as “white codes” and “green codes” [2] are cared in the PACS room service and only the patients classified as “yellow” and “red codes” are forwarded to ER for further assistance, which may include hospitalization depending on the clinical conditions.

The rotations of pediatricians in PACS include eight hours working periods, divided in two daily segments. Typically, 9 am to 12 pm, and 4-7 pm.

## Findings

**Fig. 1:**
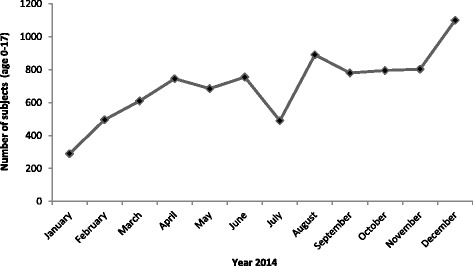
Monthly distribution of accesses to the PACS room services during the year 2014. Monthly distribution of the 8439 accesses (<18 years of age) to the PACS room services in the 5 hospitals of ASL-BT participating to the PACS program during the year 2014

During 2014 the PACS program covered a total of 3.300 h of medical service, involving all the 5 provincial public hospitals managed by the ASL-BT. The total population resident in the BAT province and served by the ASL-BT in 2014 was 392.446 [4] of which 76.563 (19.5 %) was <18 years of age. The total admissions to the PACS service rooms during a 12 months period has been 8.439, including multiple admission (Fig. 1).

**Fig. 2:**
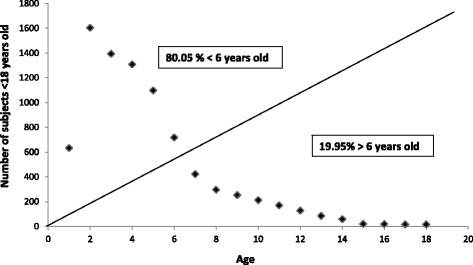
Age of subjects <18 years old admitted to the PACS rooms during 2014. Age of subjects <18 years old admitted to the PACS rooms of the 5 ASL-BT hospitals participating to the PACS program during 2014

**Fig. 3:**
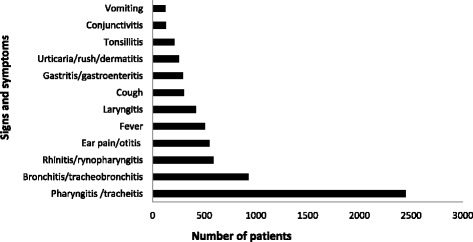
Main signs and symptoms showed by 80 % of the patients admitted to the hospital PACS room services. Main signs and symptoms shown by 80 % (6756) of the 8439 subjects <18 years old admitted to the PACS room services of five hospital participating to the PACS program during the year 2014. The remaining 20 % (1683) presented various different minor symptoms

The mean age of patients <18 years old cared for in the PACS rooms was between 3.39 and 4.41, and 80.05 % of them were <6 years old (Fig. 2). Figure 3 summarizes the twelve more frequent medical conditions observed in children upon their admission to the hospital PACS rooms.

During 2014 the total admissions to the ER departments was reduced by 14 %. In particular, the total cases registered as white and green codes in the ER Departments of the five hospitals included in PACS program were reduced by 54 %, and the number of subjects <18 year of age, hospitalized after their initial admission as white and green codes was significantly reduced by 18 % (*p* < 0.001) compared to the previous year (2013).

Finally, the PACS program has generated a significant cost containment in hospitalization costs. In fact, considering that the reported mean daily unit cost of hospitalization in Italy [5] is Euro 612/day, the saving based on a 18 % reduction of admissions generated by the SCAP program has been 110.160,00 euro/1000 hospitalizations/year. Taken together the findings reported suggest PACS to be a positive and cost effective healthcare program. PACS may be considered at national and international level as a useful model for planning future health care programs, aimed at establishing innovative yet efficient managerial solutions able to rationalize the resources not weakening the quality of services provided to the population
